# Optimizing breast and chest wall treatment planning: Integrating dynamic collimator rotation with static‐angle modulated ports in VMAT radiotherapy

**DOI:** 10.1002/acm2.70442

**Published:** 2026-02-08

**Authors:** Emily Hubley, Yin Gao, Brandon Koger, Taoran Li, Kevin Risolo, Michael Salerno, Ryan M. Scheuermann, Lei Dong, Boon‐Keng Kevin Teo

**Affiliations:** ^1^ Department of Radiation Oncology, Perelman School of Medicine University of Pennsylvania Philadelphia Pennsylvania USA; ^2^ Department of Medical Affairs Varian Medical Systems USA

**Keywords:** breast, chest wall, IMRT, OAR sparing, treatment planning, VMAT

## Abstract

**Purpose:**

RapidArc Dynamic (RAD) integrates static‐angle modulated ports (STAMPs) and a dynamic collimator into arc delivery. The optimal use of RAD, including the ideal number of STAMPs, the best use of the dynamic collimator, and the ideal relative weighting between arc and STAMPs, has yet to be reported. We aim to investigate optimized utility of these parameters for breast and chest wall treatment planning to achieve superior dosimetric results.

**Methods:**

Thirteen breast and chest wall patients were planned using RAD. Plans were created using the three different dynamic collimator options, five different arc/STAMP weighting options, and with 2, 4, and 6 STAMPs. All plans were created with automated skin flash. RAD plans were compared to conventional RapidArc (RA) plans. The DVH metrics and MUs for each plan were recorded, and a paired T‐test was used to test for statistically significant (*p* ≤ 0.05) differences between the plans.

**Results:**

“Optimize between static angles” was the best option for dynamic collimator setting. Increasing the number of STAMPs from 2 to 4 or 6 lowered PTV V105% in patients where the PTV V105% was high but provided limited benefit in most patients. Selecting arc‐dominant weighting yields significantly worse DVH metrics than a balanced weighting. Dosimetric differences were minimal between (0) Balanced, (1) Static, or (2) Static‐Dominant weighting.

**Conclusions:**

The following are recommended as a starting point for breast and chest wall RAD plans: 2 STAMPs positioned similar to breast tangents, “optimize between static angles” for the dynamic collimator, and a weighting of either (0) balanced, (1) static, or (2) static‐dominant. The arc‐dominant setting resulted in plans of the lowest quality.

## 1 INTRODUCTION

Radiation therapy (RT) has been shown to reduce both recurrence and breast cancer mortality for patients who have received breast‐conserving surgery^1^ and for node‐positive patients who have received mastectomy.^2^ Conventionally, breast and chest wall RT is delivered using 3D conformal treatment planning techniques, but intensity modulated radiation therapy (IMRT) and volumetric modulated arc therapy (VMAT) can both provide dosimetric advantages in some patients.^3^, ^4^, ^5^ Hybrid VMAT‐IMRT and VMAT‐3D methods have also been shown in the literature to improve dosimetry.^6^, ^7^

RapidArc Dynamic (RAD) solution is a new optimizer and treatment delivery technique (Varian Medical Systems, Palo Alto, USA).^8^, ^9^, ^10^, ^11^ RAD integrates static‐angle modulated ports (STAMPs) within arc delivery. During delivery, the gantry pauses at user‐selected angles to deliver monitor units via STAMPs. The MLC sequences for the arc and STAMPs are optimized simultaneously. In addition to selecting arc span, the treatment planner selects the number (minimum of 1, and maximum of 10) and location of STAMPs and the weighting of the STAMP MU relative to the arc MU. There are five options for arc versus STAMP weighting: (−2) Arc‐Dominant, (−1) Arc, (0) Balanced, (1) Static, or (2) Static‐Dominant. For a given weighting, each STAMP will have a fixed number of control points (CP). Static‐dominant weighting assigns the highest number of CP per STAMP. The overall weighting of monitor units delivered during arc versus STAMP will depend on the weighting, number or ports, arc length, and any avoidance sectors used. RAD also introduces a dynamic collimator feature where the planner has three options: 1. Static: the collimator angle is fixed throughout the entire arc, as is the case in standard VMAT, 2. Optimize between static angles: a collimator angle is selected for each STAMP and the optimizer dynamically rotates the collimator between STAMPs, or 3. Optimize: the optimizer dynamically rotates the collimator angle for all control points in the arc/ports. When the collimator is dynamically rotated between static ports or for all control points, the optimizer fits the collimator to the projection of the target(s) at each control point. Changes in collimator angle between adjacent control points are small to ensure smooth delivery throughout the arc and STAMP delivery.

In VMAT for breast and chest wall, skin flash can be added using a virtual bolus technique.^12^ RAD contains an automatic skin flash tool (ASF) which integrates skin flash directly into the optimization window, eliminating the need for manual and iterative virtual bolus techniques. Planners must select the thickness and Hounsfield Unit (HU) assignment of the ASF.^9^, ^10^

As this technology is new, no published literature exists on how to best utilize the new optimizer for treatment planning. In addition, there are many combinations in using these different options; it is not trivial to evaluate the optimal use of them. This work aims to explore the parameter space of number, location, and weighting of STAMPs and how to best employ the dynamic collimator angle for treatment planning of breast and chest wall patients.

## 2 MATERIALS AND METHODS

### 2.1 Patient selection

Nine left‐ and four right‐sided breast and chest wall patients were selected for this study. All patients in this study were simulated and treated using deep‐inspiration breath hold (DIBH). CT simulations were acquired between February 2018 and May 2019. Patients with regional nodal irradiation were omitted. Patients were selected to represent a range of patient anatomy including five intact breast, two implant reconstruction, two autologous flap reconstruction, and four chest walls with no reconstruction. Institutional Review Board approval was obtained. For all patients, the PTV and CTV were cropped 3 mm from the external patient contour.

### 2.2 RAD field setup

Nine RAD plans were created for each patient. For all plans, one partial arc was used (arc length = 200° to 220°). For left‐sided patients, the arc started between 290° and 310° and ended at 150°. For right‐sided, the arc started at 60° or 65° and ended at 210°. The arc starting position was selected based on individual patient anatomy to avoid beam entry through the contralateral breast.

The first two STAMPs were placed roughly to mimic typical tangent angles used in 3D planning (Figure 1). The beam's eye view was used to select a medial STAMP angle that would minimize the amount of heart and lung in the field. The lateral port was then placed 160°–176° away from the medial STAMP. Directly opposed ports were not used.

**FIGURE 1:**
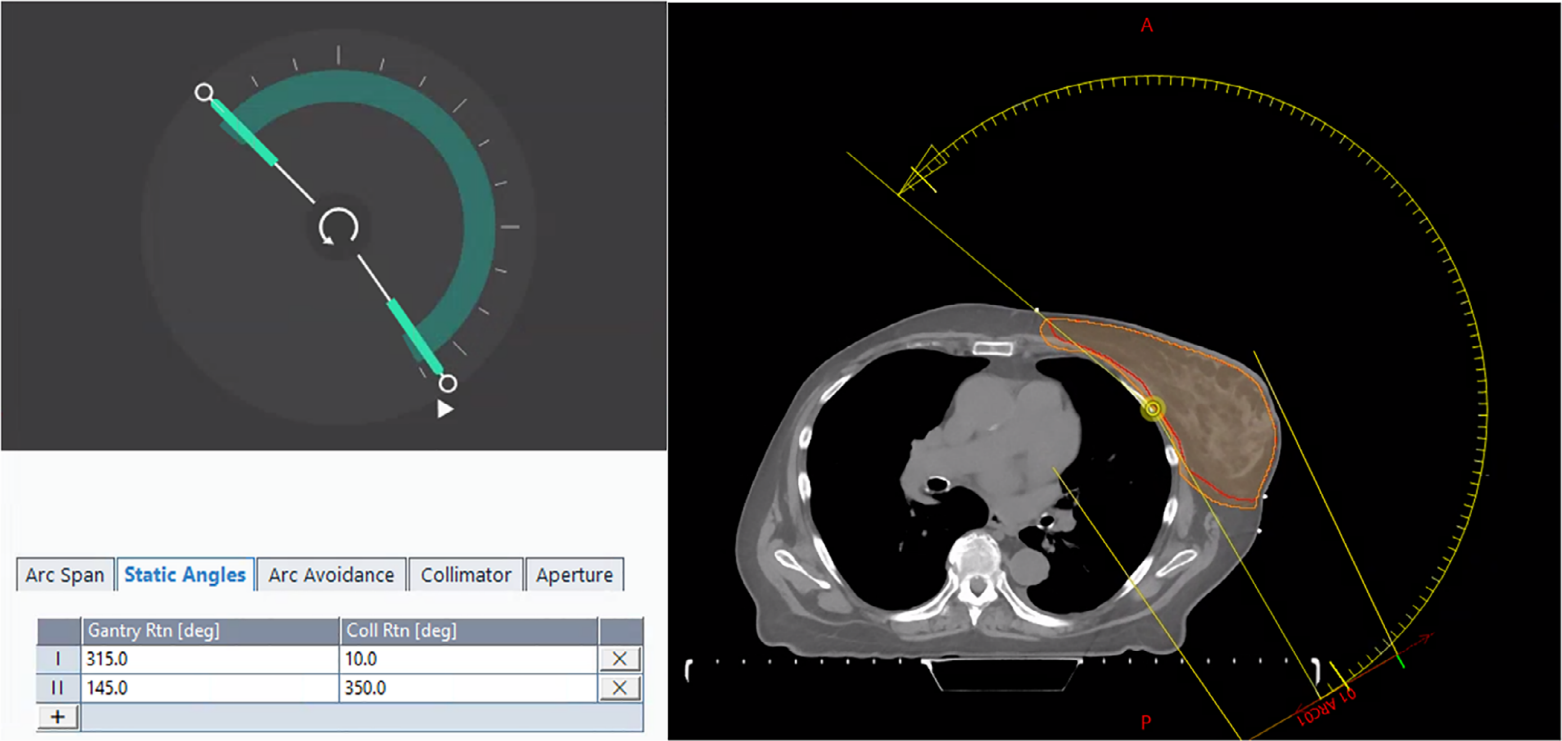
(Left) The arc span and STAMP angles are shown in teal and bright teal in the “Arc Tool” interface window. (Right) the corresponding arc span and STAMP locations are displayed on the patient. STAMP locations are indicated by the long yellow hash marks along the arc span.

In the plans using the “optimize between static angles” option for dynamic collimator, the beams eye view was used to select a collimator angle where the x jaws were parallel to the lung/PTV interface at each STAMP (Figure 2). Collimator angles ranged from 350°–355° for medial STAMPs and from 5°–10° for the lateral STAMPs. For plans with a static collimator, the collimator angle was selected based on the medial STAMP (350°–355°).

**FIGURE 2:**
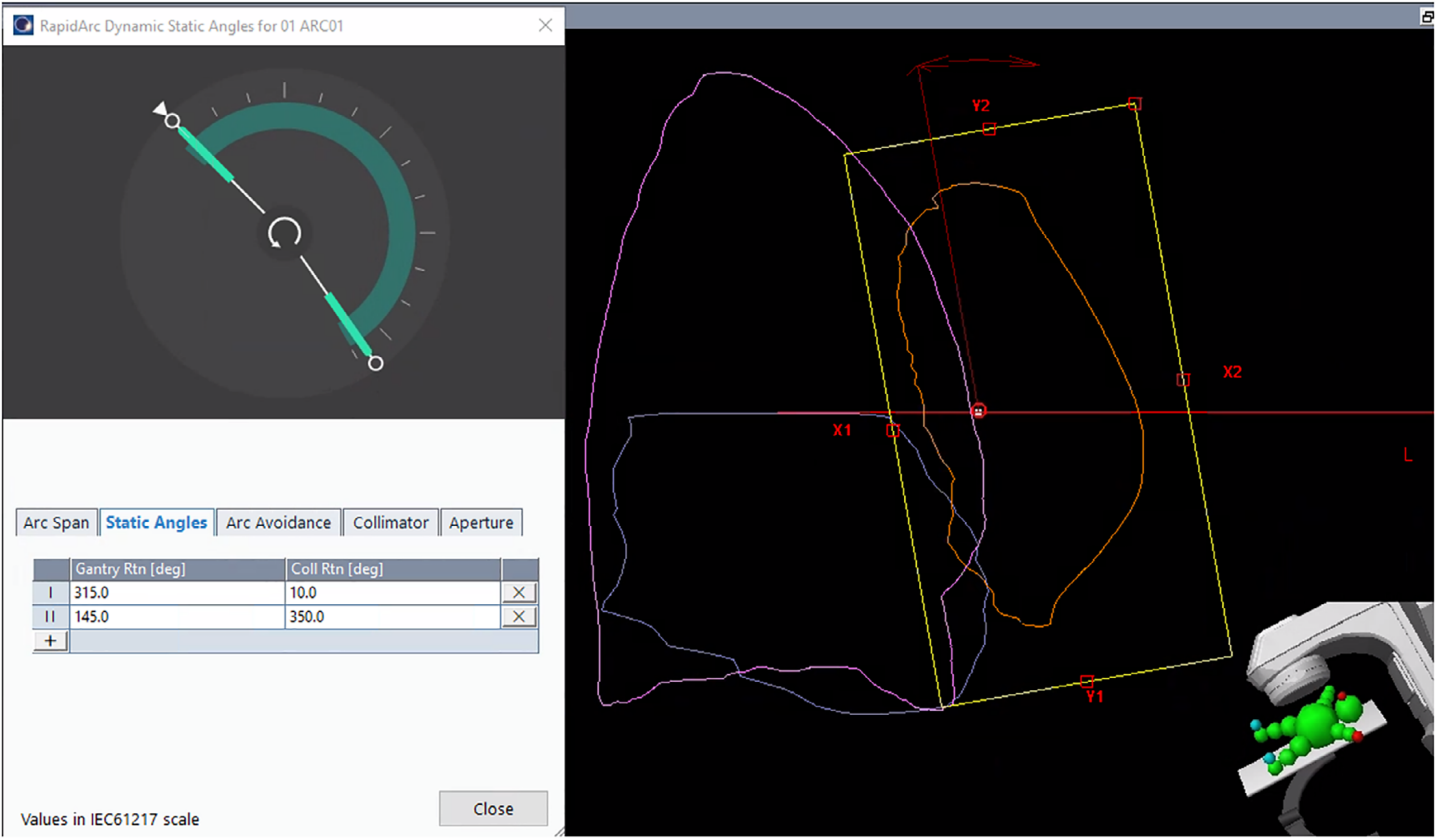
When using “optimize between static angles” dynamic collimator option, the collimator angle is selected for each STAMP in the “Static Angles” tab of the Arc Tools interface. The beam's eye view can be viewed concurrently while adjusting collimator angles. In this case, the collimator angle was selected to be roughly parallel to the PTV (orange) and ipsilateral lung (pink) interface to facilitate optimal lung (purple) and heart (blue) blocking.

All RAD plans used the automatic skin flash tool with 1 cm of skin flash and an HU assignment of −350 HU. The ASF tool allows the user to select symmetric or asymmetric skin flash; symmetric was selected for this study. The optimal thickness and HU for the ASF tool in RAD planning were investigated in a separate study,^9^ and these values align with recommendations from Lizondo et al for VMAT planning.^13^ In the RAD optimizer, the user selects the number of iterations for the optimizer to run. The possible range is 100–4000 and the default is 800. The number of iterations was set to 1000 for all RAD plans in this study to promote stable convergence with a small time cost; using a fixed iteration count across scenarios ensured a fair comparison.

The RAD plans varied the number of STAMPs, STAMP versus arc weighting, and dynamic collimator use (Table 1). The “base” RAD plan, which was used as a baseline for comparing all other plans, used 2 STAMPs, (0) Balanced arc/port weighting, and the dynamic collimator setting “Optimize between static angles” was selected. To assess the optimal number of STAMPs, the base RAD plan was copied and STAMPs were added 8° and 16° from the first two ports to create plans with a total of 4 and 6 STAMPs. The plans were re‐optimized. The base RAD plan was also copied and re‐optimized using the “Static” and “Optimize” options, and with the (−2) Arc‐Dominant, (−1) Arc, (1) Static, or (2) Static‐Dominant weighting options.

**TABLE 1:** The number of STAMPs, STAMP weighting, and dynamic collimator used.

Number of STAMPs	Dynamic collimator	Arc/STAMP weighting	Skin flash
2^*^	Optimize btw static^†^	(0) Balanced	Yes
4	Optimize btw static	(0) Balanced	Yes
6	Optimize btw static	(0) Balanced	Yes
2	Optimize btw static	(−2) Arc‐Dominant	Yes
2	Optimize btw static	(−1) Arc	yes
2	Optimize btw static	(1) Static	Yes
2	Optimize btw static	(2) Static‐Dominant	Yes
2	Static	(0) Balanced	Yes
2	Optimize	(0) Balanced	Yes

* indicates the “base” RAD plan against which all other plans were compared.

^†^ Optimize btw static = Optimize between static angles.

### 2.3 RapidArc plans

A RapidArc (RA) plan was also created for each patient with two arcs, using the same arc span as the RAD plans. The collimator was set to 350° and 10° in the two arcs. Skin flash was created manually using a virtual bolus approach.^12^ The skin flash thickness was 1 cm and HU was set to −350 HU.

### 2.4 Optimization

For each patient, both RAD and RA plans were initialized and evaluated using the same institutional clinical objectives (Table 2). The RAD optimization workflow is essentially the same as conventional RA, with the primary difference being the optimizer. Conventional RA optimizes the MLC sequence, gantry speed, and dose rate for the arc. In contrast, the RAD optimizer simultaneously optimizes MLC sequences for the arc and user‐selected static‐angle STAMPs while also honoring the chosen dynamic collimator option. In conventional RA, objectives may be modified during optimization runs based on planner discretion and clinical priorities. However, planning objectives are fixed during each RAD optimization run and cannot be adjusted interactively. Planners performed multiple optimization runs as needed to achieve clinically acceptable plans. This design holds clinical goals constant and isolates differences arising from the inherent planning workflow. The ‘number of iterations’ parameter in RAD controls the internal update steps of the optimizer, functioning as a convergence budget. We used 1000 iterations for all RAD plans to ensure convergence. Because RAD utilizes a GPU‐based optimizer, the overall optimization process remains efficient despite the absence of live adjustment.

**TABLE 2:** Treatment planning objectives for breast and chest wall RT at our institution.

Structure	Objective	Variation	Clinical priority^†^
PTV	D95% ≥ 38.05 Gy	≥ 36.05 Gy	1
PTV	V36.05 Gy ≥ 99%	≥ 98%	1
PTV	V105% ≤10%	≤ 15%	1
Heart (left‐sided)	*D*_mean_ < 2 Gy	< 5 Gy	2
Heart (right‐sided)	*D*_mean_ < 1 Gy	< 5 Gy	2
Ipsilateral lung	*D*_mean_ < 7 Gy	< 13 Gy	2
Ipsilateral lung	V16 Gy ≤ 15%	≤ 20%	2
Ipsilateral lung	V4 Gy ≤ 35%	≤ 50%	2
Contralateral lung	*D*_mean_ < 1 Gy	< 2.5 Gy	3
Contralateral lung	V40 Gy ≤ 10%	≤ 15%	3
Contralateral breast	*D*_mean_ < 1 Gy	< 3 Gy	4

^†^ 1 indicates the highest priority and 4 indicates the lowest priority for dosimetric evaluation.

All RA and RAD plans were created with a prescription dose of 4005 cGy in 15 fractions and optimized to achieve clinically acceptable plans. Following optimization, all plans were normalized to ensure PTV D95% = 95%. Dose calculations for both RA and RAD plans were performed using Acuros v18.1.0 in Eclipse v18.1 to ensure consistency. The number of monitor units (MU) for each plan was recorded. All RA and RAD plans were reviewed by the clinical team for quality and were deemed clinically acceptable prior to analysis.

A paired T‐test was used to compare the target and organ at risk (OAR) dose‐volume histogram (DVH) parameters in all plans to those in the base RAD plan and those in the RA plan. A *p*‐value ≤ 0.05 indicates statistical significance. Given the small sample size and heterogeneity, statistical tests are exploratory. We primarily report descriptive metrics (mean ± standard deviation) and provide comparisons as context only. These analyses are not powered for confirmatory inference.

### 2.5 Timing study

Once optimization was completed, the planner copied the RA plan and the base RAD plan and re‐ran the optimization and calculation without changing any optimization objectives, for the purposes of a timing study. The times for a single optimization and dose calculation were recorded.

## 3 RESULTS

### 3.1 Number of STAMPs

Table 3 displays results comparing plans with 4 and 6 STAMPs compared to the base RAD plan and the RA plan. The bolded values indicate a statistically significant (*p* ≤ 0.05) difference from the base RAD plan; the underlined values indicate a statistically significant (*p* ≤ 0.05) difference from the RA plan. Increasing the number of STAMPs reduced hotspots in the target, decreasing the mean PTV V42.05 Gy (V105%). Other DVH metrics were largely unchanged. Any number of STAMPs resulted in similar or decreased OAR doses compared to the RA plan though most differences were not statistically significant.

**TABLE 3:** DVH metrics for the RA plan, and RAD plans with 2, 4, and 6 STAMPs.

			Weighting: balanced Collimator: “optimize between static angles”		
		RA	2 ports (base RAD plan)	4 ports RAD	6 ports RAD
PTV	D95% (Gy)	38.0	38.0	38.0	38.0
PTV	V36.05 Gy (%)	99.4 ± 0.2	99.5 ± 0.3	99.5 ± 0.3	99.5 ± 0.3
PTV	V42.05 Gy (%)	4.2 ± 3.3	2.4 ± 4.0	**1.1 ± 2.1**	**0.7 ± 1.3**
Heart	D_mean_ (Gy)	1.4 ± 0.4	1.4 ± 0.4	1.4 ± 0.5	1.4 ± 0.5
Ipsi Lung	D_mean_ (Gy)	6.3 ± 0.7	5.9 ± 1.1	6.0 ± 1.0	5.9 ± 0.9
Ipsi Lung	V16Gy (%)	13.5 ± 2.2	13.1 ± 3.7	13.1 ± 3.4	12.8 ± 3.2
Ipsi Lung	V4Gy(%)	**33.9 ± 2.9**	30.1 ± 5.3	30.5 ± 4.7	30.1 ± 4.5
Contra Lung	D_mean_ (Gy)	1.0 ± 0.2	0.7 ± 0.3	0.8 ± 0.3	0.8 ± 0.3
Contra Lung	V4Gy (%)	0.7 ± 1.0	0.9 ± 1.4	1.0 ± 1.0	1.2 ± 1.2
Contra Breast	D_mean_ (Gy)	1.1 ± 0.3	1.0 ± 0.4	1.0 ± 0.4	1.0 ± 0.5
	MU	**574 ± 63**	828 ± 76	**856 ± 54**	**888 ± 53**

### 3.2 Arc vs. STAMP weighting

Table 4 compares plans with different arc vs. STAMP weighting to the base RAD and RA plans. The bolded values indicate significantly different (*p* ≤ 0.05) results than the base RAD plan; the underlined values indicate statistically significant (*p* ≤ 0.05) difference from the RA plan. All DVH metrics except for ipsilateral lung V16Gy were significantly worse in the (−2) Arc‐Dominant plans than in the (0) Balanced plans. The PTV V42.05 Gy, heart mean, ipsilateral lung V4Gy, and contralateral breast were all reduced (or unchanged) as the STAMP weighting was increased.

**TABLE 4:** DVH metrics for the RA, base RAD plan (weighting = 0) and plans other arc/port weighting options.

			2 ports, collimator: “optimize between static angles”				
		RA	(−2) Arc dominant	(−1) Arc	(0) Balanced (base RAD plan)	(1) Static	(2) Static dominant
PTV	D95% (Gy)	38.0	38.0	38.0	38.0	38.0	38.0
PTV	V36.05 Gy (%)	99.4 ± 0.2	**99.3 ± 0.4**	99.5 ± 0.3	99.5 ± 0.3	99.5 ± 0.3	99.6 ± 0.3
PTV	V42.05 Gy (%)	4.2 ± 3.3	**10.9 ± 8.1**	2.3 ± 2.9	2.4 ± 4.0	1.9 ± 3.6	1.1 ± 1.9
Heart	*D*_mean_ (Gy)	1.4 ± 0.4	**1.7 ± 0.6**	1.4 ± 0.5	1.4 ± 0.4	1.3 ± 0.4	1.3 ± 0.4
Ipsi lung	*D*_mean_ (Gy)	6.3 ± 0.7	**6.3 ± 1.2**	5.9 ± 1.1	5.9 ± 1.1	5.9 ± 1.2	5.9 ± 1.1
Ipsi lung	V16Gy (%)	13.5 ± 2.2	13.5 ± 3.3	**12.8 ± 3.6**	13.1 ± 3.7	13.2 ± 4.0	13.1 ± 3.8
Ipsi lung	V4Gy(%)	**33.9 ± 2.9**	**34.4 ± 7.1**	31.0 ± 5.5	30.1 ± 5.3	29.9 ± 5.2	29.7 ± 5.2
Contra lung	*D*_mean_ (Gy)	1.0 ± 0.2	**1.1 ± 0.3**	0.9 ± 0.5	0.7 ± 0.3	0.8 ± 0.3	0.8 ± 0.3
Contra lung	V4Gy (%)	0.7 ± 1.0	**3.2 ± 2.8**	1.9 ± 3.4	0.9 ± 1.4	0.9 ± 1.3	1.0 ± 1.2
Contra breast	*D*_mean_ (Gy)	1.1 ± 0.3	**1.2 ± 0.4**	**1.1 ± 0.4**	1.0 ± 0.4	1.0 ± 0.4	1.0 ± 0.4
	MU	**574 ± 63**	**787 ± 77**	802 ± 96	828 ± 76	841 ± 74	**856 ± 68**

### 3.3 Dynamic collimator use

The DVH parameters resulting from the three dynamic collimator options are listed in Table 5. The bolded values indicate significantly different (*p* ≤ 0.05) results than the RAD base plan (optimize between static angles); the underlined values indicate statistically significant (*p* ≤ 0.05) difference from the RA plan. No DVH metrics were significantly improved over the base RAD plan with fully static or fully optimized collimator options were selected. Most metrics were similar.

**TABLE 5:** The mean ± standard deviation DVH metrics for the three dynamic collimator options.

			Number of ports: 2, Weighting: balanced		
		RA	Optimize between static angles (base RAD plan)	Static	Optimize
PTV	D95% (Gy)	38.0	38.0	38.0	38.0
PTV	V36.05 Gy (%)	99.4 ± 0.2	99.5 ± 0.3	99.5 ± 0.4	99.6 ± 0.3
PTV	V42.05 Gy (%)	4.2 ± 3.3	2.4 ± 4.0	3.3 ± 4.4	1.3 ± 2.0
Heart	*D*_mean_ (Gy)	1.4 ± 0.4	1.4 ± 0.4	1.2 ± 0.4	1.3 ± 0.5
Ipsi lung	*D*_mean_ (Gy)	6.3 ± 0.7	5.9 ± 1.1	**6.1 ± 1.1**	5.9 ± 1.1
Ipsi lung	V16Gy (%)	13.5 ± 2.2	13.1 ± 3.7	**13.7 ± 3.7**	12.8 ± 3.5
Ipsi lung	V4Gy(%)	**33.9 ± 2.9**	30.1 ± 5.3	31.1 ± 4.6	**31.3 ± 5.4**
Contra lung	*D*_mean_ (Gy)	1.0 ± 0.2	0.7 ± 0.3	0.6 ± 0.3	0.8 ± 0.3
Contra lung	V4Gy (%)	0.7 ± 1.0	0.9 ± 1.4	0.6 ± 1.5	0.9 ± 1.2
Contra breast	*D*_mean_ (Gy)	1.1 ± 0.3	1.0 ± 0.4	0.9 ± 0.5	1.0 ± 0.4
	MU	**574 ± 63**	828 ± 76	**786 ± 62**	836 ± 75

### 3.4 Timing study

For RA plans, the mean ± standard deviation of optimization and calculation time are 307 ± 98 s and 19 ± 4 s (total optimization + calculation time = 326 ± 98 s). For RAD plans, the optimization and calculation times were 63 ± 9 s and 11 ± 2 s (total optimization + calculation time = 74 ± 9 s).

## 4 DISCUSSION

Increasing the number of STAMPs decreased the PTV V42.05 Gy. For most patients, this decrease was <1.5% when 2, 4, or 6 STAMPs were used. However, for two patients, the PTV V42.05 Gy dramatically decreased from 9.8% to 5.7% to 3.6% and from 12.6% to 5.8% to 0.4% when the number of STAMPs was increased from 2 to 4 to 6. These two patient anatomies were both chest wall with no reconstruction, however other chest wall patient anatomies did not have high heterogeneity. Changing the number of STAMPs did not significantly change other DVH parameters. It is therefore recommended to start planning with two STAMPs and increase the number if the target heterogeneity is too high.

Arc‐Dominant (−2) weighting resulted in significantly higher DVH metrics than the base RAD plan. Most metrics were reduced or unchanged as the weighting increased from (0) Balanced to (2) Static‐Dominant. Based on all DVH metrics examined in this study, a (0) Balanced, (1) Static, or (2) Static‐Dominant weighting is recommended when two STAMPs are used. In general, increasing the weighting of the STAMPs decreases the low‐dose spill which is reflected in the DVH metrics.

The fully static or fully optimized collimator options did not result in any significant improvement in any OAR doses compared to the “optimize between static angles” base RAD plan. With fully static collimator angle, the ipsilateral lung mean dose and V16Gy were both significantly higher than the base RAD plan. With fully optimized dynamic collimator, the ipsilateral lung V4Gy was significantly higher than the base RAD plan. A fully optimized dynamic collimator provided the highest PTV V36.05 Gy and lowest PTV V42.05 Gy. With this setting, the optimizer selects collimator angles at each control point to maximize the volume of the target within the beam's eye view/jaws aperture and does not take into account the positions of the OARs or optimal collimator angle to allow MLC blocking of OARs. Using a static collimator provided the lowest MUs.

This study investigated a relatively small cohort of patients with heterogeneous target volumes including intact breasts, implant or flap‐based reconstructed breasts, and postmastectomy chest walls with no reconstruction. The PTVs ranged from 208 to 986cc. As such, the recommendations will need to be tailored for each individual patient. This cohort of patients also excluded any regional lymph node treatment volumes, so the results may not be applicable to this patient population. Because of the small patient cohort, statistical analysis is not powered for significance. All plans were created with 1 cm of automatic skin flash. The amount of skin flash used clinically will vary by patient and institution and must take into account the patient anatomy, motion management used, and the daily image guidance used. The number of MU in each plan will affect the treatment delivery time which is especially relevant for patients treated with DIBH. The delivery time of the plans was not measured in this work.

Compared to the RA plans, most metrics were similar or improved in all RAD plans. The larger improvements were observed in the low‐dose metrics.

Our approach compares RA and RAD under identical clinical goals while respecting each modality's intrinsic workflow (interactive retuning in RA vs. per‐run updates in RAD). In this way, dosimetric differences reflect the planning mode rather than divergent targets or dose engines. For efficiency, we reported compute time per run (optimizer + intermediate dose calculation), an operator‐independent but reproducible measure of optimizer speed that avoids operator dependent variability in total time to acceptance.

The timing study results indicate markedly reduced optimization times for RAD compared to RA plans. The magnitude of this reduction will depend on the user's hardware. Further, this study quantified compute time (optimizer + dose calculation) under controlled conditions to avoid confounding by operator behavior. ‘Time to a clinically acceptable plan’ depends on planner experience, template maturity, and institutional workflow and was therefore not measured. Nevertheless, the markedly shorter compute time with RAD suggests potential end‐to‐end efficiency benefits.

## 5 CONCLUSIONS

When planning breast and chest wall patients with RAD, a plan with two STAMPs approximating tangent angles provided the best dosimetric parameters. Increasing the number of STAMPs from 2 to 4 or 6 can reduce the PTV V105% in patients where the PTV V105% is large, but for most patients in this cohort, 2 STAMPs was sufficient. Turning on “optimize between static angles” for the dynamic collimator, and setting the collimator angle at each STAMP to allow for optimal heart and lung blocking resulted in improved plans. For arc/STAMP weighting, (0) Balanced, (1) Static, or (2) Static‐Dominant achieved the best plans. (1) Static or (2) static‐dominant can reduce the PTV V105% in patients with high PTV V105%. (−2) Arc‐Dominant weighting increased most OAR DVH metrics and therefore is not recommended.

## AUTHOR CONTRIBUTIONS

All the above listed authors have contributed directly to the intellectual content, preparation, and final review of this manuscript.

## CONFLICT OF INTEREST STATEMENT

Emily Hubley and Michael Salerno have received honoraria from Varian Medical Systems for speaking about RapidArc Dynamic. Taoran Li is an employee of Varian Medical Systems.
